# Unveiling the neuroprotective potential of dietary polysaccharides: a systematic review

**DOI:** 10.3389/fnut.2023.1299117

**Published:** 2023-11-22

**Authors:** Rui Guo, Jingxi Pang, Junhe Zhao, Xiao Xiao, Jing Li, Jingmeng Li, Wenxiu Wang, Shuang Zhou, Yu Zhao, Zilong Zhang, Hongwang Chen, Tian Yuan, Shan Wu, Zhigang Liu

**Affiliations:** ^1^Laboratory of Functional Chemistry and Nutrition of Food, College of Food Science and Engineering, Northwest A&F University, Yangling, Shaanxi, China; ^2^Northwest A&F University Shenzhen Research Institute, Shenzhen, Guangdong, China; ^3^National Center of Technology Innovation for Dairy, Hohhot, Inner Mongolia, China; ^4^Research and Development Center, Xi'an Yinqiao Dairy Technology Co., Ltd., Xi'an, Shaanxi, China

**Keywords:** dietary polysaccharides, neuroprotection, central nervous system disorders, antioxidant, anti-inflammatory, gut microbiota, gut-brain axis

## Abstract

Central nervous system (CNS) disorders present a growing and costly global health challenge, accounting for over 11% of the diseases burden in high-income countries. Despite current treatments, patients often experience persistent symptoms that significantly affect their quality of life. Dietary polysaccharides have garnered attention for their potential as interventions for CNS disorders due to their diverse mechanisms of action, including antioxidant, anti-inflammatory, and neuroprotective effects. Through an analysis of research articles published between January 5, 2013 and August 30, 2023, encompassing the intervention effects of dietary polysaccharides on Alzheimer’s disease, Parkinson’s disease, depression, anxiety disorders, autism spectrum disorder, epilepsy, and stroke, we have conducted a comprehensive review with the aim of elucidating the role and mechanisms of dietary polysaccharides in various CNS diseases, spanning neurodegenerative, psychiatric, neurodevelopmental disorders, and neurological dysfunctions. At least four categories of mechanistic bases are included in the dietary polysaccharides’ intervention against CNS disease, which involves oxidative stress reduction, neuronal production, metabolic regulation, and gut barrier integrity. Notably, the ability of dietary polysaccharides to resist oxidation and modulate gut microbiota not only helps to curb the development of these diseases at an early stage, but also holds promise for the development of novel therapeutic agents for CNS diseases. In conclusion, this comprehensive review strives to advance therapeutic strategies for CNS disorders by elucidating the potential of dietary polysaccharides and advocating interdisciplinary collaboration to propel further research in this realm.

## 1 Introduction

Central nervous system (CNS) disorders are a growing and costly global health problem. According to the World Health Organization (WHO), CNS disorders account for more than 11% of the overall disease burden in high-income countries (1). These diseases not only affect the life quality of patients but also have a major impact on families, society, and global healthcare resources.

Concerning the high incidence and sophisticated outcome of CNS disorders, pharmacological intervention, surgical resection, and brain stimulation therapy are currently commonly used (2, 3). Despite recent advances in these treatments, many patients continue to experience relevant symptoms that significantly impact their work, relationships, and self-care (4, 5). Hence, scientific researchers are still continuously seeking more effective therapeutic strategies to reduce the burden of these diseases. In recent years, natural components of plants have sparked extensive research interest as a compelling intervention (6). Among them, polysaccharides, as a complex and diverse class of biomolecules, have a significant potential for the intervention of neurological disorders, and their multiple mechanisms of action, such as antioxidant, anti-inflammatory, and neuroprotective, offer new perspectives on therapeutics (6, 7).

By retrieving research articles about the neuroprotective effects of dietary polysaccharides published from 2013 to 2023 on databases such as PubMed, Web of Science, and Google Scholar, it was found that researchers have widely focused on Alzheimer’s disease, Parkinson’s disease, depression, anxiety disorders, autism spectrum disorder, stroke, and epilepsy. Although these diseases cover multiple fields such as neurodegenerative diseases, psychiatric disorders, neurodevelopmental disorders, and neurological dysfunctions, there is a lack of systematic reviews exploring the protective effects and related mechanisms of dietary polysaccharides against CNS diseases.

In this sense, the present review aims to comprehensively elucidate the potential roles, intrinsic mechanisms, and future research directions of dietary polysaccharides in various CNS diseases. Through this review, we hope to provide an in-depth and comprehensive research report for the nutritional disciplines to explore the prospect of dietary polysaccharides as potential therapeutic approaches for a wide range of CNS disorders. At the same time, we encourage interdisciplinary collaborations to promote in-depth research in these areas and make greater contributions to improving patients’ life quality and advancing medical science.

## 2 Polysaccharides: structures, sources, and biological activities

Polysaccharides are vital macromolecules found abundantly in nature, and serve as fundamental components in the cell walls of plants, fungi, and bacteria, providing structural support and protection (8). In addition, polysaccharides act as an essential energy storage form in plants and animals, offering a sustained and readily available energy source (9, 10). Polysaccharides can be categorized based on their sources, including plants, animals, microorganisms, and edible fungi. Plant-derived polysaccharides originate from various plants, encompassing medicinal herbs, fruits, and vegetables, and can be extracted from roots, stems, leaves, and fruits, or may naturally exist in plants, such as gum and cellulose. Animal-derived polysaccharides, are sourced from animal tissues, for instance, chondroitin sulfate from squid cartilage. Microbial-derived polysaccharides are obtained from microorganisms, such as Lactobacillus polysaccharides and yeast β-glucans. Additionally, there are polysaccharides derived from edible fungi, such as mushrooms, black fungus, and Ganoderma lucidum.

Polysaccharides are typically composed of more than ten repeating units of monosaccharides, such as glucose, fructose, or mannose, linked together in various configurations (11). The specific arrangement of these sugar units, as well as the presence of side chains and branching, contribute to the unique physicochemical properties and biological activities exhibited by polysaccharides (12). In recent years, there has been a growing interest in the study of dietary polysaccharides derived from plants, animals, and microorganisms, which have long been recognized for their valuable contributions to human health and nutrition, as they are believed to exhibit a wide range of biological activities (12).

Dietary polysaccharides have been shown to possess a wide array of health-promoting effects, including immunomodulatory, antioxidant, antitumor, anti-inflammatory, and antimicrobial activities. These bioactivities make them potential candidates for the development of natural medicines, functional foods, and nutraceuticals. Moreover, their ability to regulate various physiological processes, such as oxidative stress, lipid regulation, and gut microbiota modulation, highlights their potential in the prevention and management of CNS disorders (11). The exploration and biological evaluation of dietary polysaccharides have gained significant momentum in recent years, driven by advancements in extraction and purification techniques, like functional factors from the traditional Chinese medicine, marine organisms, edible fungus, and others. The study of dietary polysaccharides represents a fascinating and promising area of research, offering numerous possibilities for the development of novel therapeutic agents and functional foods.

## 3 Dietary polysaccharides and the interventions for Parkinson’s disease

Parkinson’s disease (PD) is a prevalent neurodegenerative movement disorder that commonly affects middle-aged and elderly individuals, characterized by clinical manifestations such as resting tremor, motor retardation, myotonia, and postural gait abnormalities (13). The major pathological features include the degenerative loss of dopaminergic neurons in the nigrostriatal circuit and the formation of Lewy bodies in multiple brain regions (14). As of 2015, the prevalence of PD in individuals aged over 60 years in China was 1.37% (15), in 2019, worldwide data disclosed over 8.5 million individuals were living with PD, the surge in disability and fatality rates due to PD outpaces all other neurological diseases, imposing a substantial burden on families and society for treatment (16). Currently, there is no cure for PD. Drugs such as Levodopa, Pramipexole, and others are commonly used to increase the level of dopamine or imitate its effects to improve or alleviate symptoms, however, long-term use can result in significant adverse reactions or varying degrees of reduced efficacy (17, 18).

PD’s etiology and pathogenesis remain unclear, but oxidative stress caused by reactive oxygen species (ROS) imbalance in mitochondria is closely implicated in the selective degeneration of dopaminergic neurons in the substantia nigra (19). It is known that dietary polysaccharides act as free radical scavengers, thus protecting mitochondria from oxidative damage and contributing to the normal function and health of the organism, which can be a practical strategy for preventing PD progression (20). Research has demonstrated that dietary polysaccharides (including *Grifola frondosa* extract (21), low molecular weight chitosan (22), inulin (23), fucoidan (24), astragalus polysaccharides (25), glycosaminoglycans (26), and polysaccharides from the starfish (27)) can significantly reduce ROS levels in animal or cell models of PD while preserving mitochondrial functionality, such as restoring mitochondrial membrane potential (ΔΨm), enhancing mitochondrial respiratory function, and increasing mitochondrial complex enzyme activity. Moreover, polymannuronic acid (28) can alleviate neuroinflammation caused by oxidative stress in PD mouse models. Notably, PD is pathologically based on the death of dopaminergic neurons leading to a reduction in dopamine levels (29). The intervention studies on dietary polysaccharides for PD focus on their direct effects on neural cells. It has been found that in rodent models of PD, polymannuronic acid administration prevented the loss of dopaminergic neurons in the substantia nigra pars compacta (28). Fucoidan mitigated the degeneration of dopaminergic neurons via the peroxisome proliferator-activated receptor gamma coactivator 1-alpha (PGC-1α) and nuclear transcription factor 2 (Nrf2) pathway (24). Astragalus polysaccharides and *Ganoderma lucidum* polysaccharides both enhanced the proportion of tyrosine hydroxylase-positive cells closely associated with dopaminergic neurons (25, 30).

In 2003, Braak first hypothesized that PD originated in the gastrointestinal tract, proposing that an unproven pathogen or toxin destroying the gastrointestinal mucosal barrier may cause α-synuclein to misfold and deposit in the enteric nerve plexus, and travel retrograde along the posterior intestinal neurons and vagus nerve into the CNS (31). Subsequently, a growing body of research has revealed the profound impact of abnormal microbiota and its metabolites on the pathogenesis and clinical manifestations of PD. Therefore, the regulation of gut microbiota represents another potential intervention method for PD. Polymannuronic acid from brown seaweed polysaccharide treatment caused changes in the gut microbial composition and dramatic changes in the digestion and metabolism of dietary protein and fat, leading to an increase in the content of short-chain fatty acids (SCFAs) in the feces of PD mice (28). Inulin intake, improved the abundance of *Bifidobacterium* and *Lactobacillus*, which are strongly positively associated with behavior in a model of gestational exposure to PD toxin (23).

Therefore, diverse dietary polysaccharides showed promise in reducing ROS and preserving mitochondria in PD models. Meanwhile, the effect of dietary polysaccharides on the gut microbiota may be a new avenue of intervention in PD. All these studies showed that dietary polysaccharides can be promising tools in the control of PD (Table 1).

**Table 1:** Recent studies on neuroprotective effects of dietary polysaccharides on PD model.

Type of dietary polysaccharides	Models/methods	Protective effects	Authors, references
Glycosaminoglycans (shark cartilage chondroitin 6-sulfate, porcine intestinal mucosa dermatan 4-sulfate, dermatan 2,6-disulfate from viscera of ascidian *Phallusia nigra*)	Murine neuroblastoma cell line neuro 2A / 0.025, 0.05, 0.1, 0.2, and 0.4 μg/mL added to the culture medium	Glycosaminoglycans reduced apoptosis and improved ΔΨm in murine neuroblastoma cells challenged by rotenone, showing neurogenic and neuroprotective properties.	Medeiros et al. (26)
*Grifola frondosa* extract (66.7% β-glucan)	Male and female *drosophila melanogaster* /diluted in the culture medium to 0.05% or 0.2% and supplemented lifelong	*Grifola frondosa* Extract Extended Lifespan in PD drosophila model.	Tripodi et al. (21)
Polymannuronic acid from brown seaweed polysaccharides	8-week-old male C57BL/6 J mice / 30 mg/kg oral gavage for 4 weeks	Polymannuronic acid from brown seaweed polysaccharides administration improved motor functions by preventing dopaminergic neuronal loss in PD mice model, alleviated inflammation in gut, brain and systematic circulation.	Dong et al. (28)
Low molecular weight chitosan exerts	Male *drosophila melanogaster* / 5, and 10 mg/mL in basal media for 7 days	Administration of low molecular weight chitosan reversed the locomotor impairment and exploratory deficitsin, changed the biochemical parameters to normal level and increased the survival rate in PD drosophila model.	Pramod Kumar and Harish Prashanth (22)
Inulin	Pregnant Sprague–Dawley rats / 2 g/kg oral administration from gestation 0–19 days	Inulin supplementation during pregnancy significantly improved maternal exploratory behavior and counteracted gestational rotenone-induced oxidative stress, improved AchE activity and mitochondrial ATP production, and alleviated mitochondrial dysfunction, and neurochemical changes in maternal and fetal brains.	Krishna and Muralidhara (23)
Fucoidan	Male Sprague–Dawley rats / 35, 70, and 140 mg/kg oral gavage for 38 days	Fucoidan reversed the loss of substantia nigra dopaminergic neurons and striatal dopaminergic fibers, substantia nigra mitochondrial respiratory function, decreased striatal dopamine level, ROS overformation, and behavioral defects in PD rat model.	Zhang et al. (24)
Astragalus polysaccharides	8-week-old male C57B/6 mice / 10 mg/kg oral gavage for 2 weeks	Astragalus polysaccharides attenuated motor dysfunction and high levels of ROS, and stabilized mitochondrial in PD mouse model.	Liu et al. (25)
*Ganoderma lucidum* polysaccharides	Primary mesencephalic dopaminergic cell from OF1/SPF mouse / 12.5, 25, 50 and 100 μg/mL added to the culture medium	*Ganoderma lucidum* polysaccharides inhibited cell apoptosis through suppressing oxidative stress in primary cell culture during dopaminergic neurons degeneration.	Guo et al. (30)
Polysaccharides from the starfish (glucan and sulfated mannoglucan)	Mouse dopaminergic neuronal cell lines MES 23.5 / 1 and 0.1 mg/mL added to the culture medium	The polysaccharides from the starfish scavenged hydroxyl radical and superoxide radical in PD cell model, which exhibited neuroprotective activity.	Zhang et al. (27)

## 4 Dietary polysaccharides and the interventions for Alzheimer’s disease

Alzheimer’s disease (AD) is a common neurodegenerative disease worldwide and one of the most prevalent forms of dementia in the elderly (32). As reported previously, the all-cause mortality rate for AD in individuals aged 70 years and older is 5.32% (32, 33). Currently, there are approximately 50 million reported cases of AD globally, which will be multiple times higher by 2050 as the population ages (32). The main symptoms of AD vary in severity and progression among individuals, while the most common symptoms include memory loss, cognitive decline, language and communication problems, behavioral and mood changes (33). Brain β-like amyloid (Aβ) deposition and neuronal fibrillary tangles (NFTs) formed by Tau hyperphosphorylation are considered important pathological indicators for AD (34).

Currently, there is no specific treatment for AD due to its unclear pathogenesis. In most cases, AD patients develop neurodegenerative disease as a result of genetics and environment, including apolipoprotein E genotype, metabolic syndrome, neuroinflammation, oxidative stress, and unhealthy diets (35). Oxidative stress, a common feature of neurodegenerative diseases, accelerates the progression of AD by causing mitochondrial dysfunction, neuron membrane damage, apoptosis, and neuroinflammation (36). Therefore, reducing oxidative stress with dietary polysaccharides is considered a prospective strategy to hinder AD pathology. Several studies have found *Cistanche deserticola* polysaccharides (37), *poria cocos* polysaccharides (38), non-saponin fraction with rich polysaccharides from Korean red ginseng (39), polysaccharides of *Taxus chinensis* var. *mairei* Cheng et L.K.Fu (40), astragalus polysaccharides (41, 42), *Angelica sinensis* polysaccharides (43), *Schisandra chinensis* (Turcz.) Baill. polysaccharide (44), *Inonotus obliquus* polysaccharide (45), *Flammulina velutipes* polysaccharides (46), Chondroitin sulfate E from squid cartilage (47), and low molecular weight chondroitin sulfate from shark cartilage (48) possessed the ability to inhibit oxidative stress and reduce neuroinflammation in animal models of AD. Additionally, *Alpinia oxyphylla* crude polysaccharides (49) and polysaccharides of *Schisandra* Chinensis Fructus (50) inhibited the inflammatory response in the AD mouse model and reduced the release of pro-inflammatory factors such as Interleukin-1 beta (IL-1β) and Tumor Necrosis Factor-alpha (TNF-α). Among them, *Angelica sinensis* polysaccharides (43), non-saponin fraction with rich polysaccharides from Korean red ginseng (39), polysaccharides from *Lycium barbarum* (51), *poria cocos* polysaccharides (38), *Ganoderma lucidum* polysaccharides (52) and *Codonopsis pilosula* polysaccharides (53) also reduced neuronal apoptosis, increased neuronal regeneration and restored synaptic dysfunction in some brain regions. *Angelica sinensis* polysaccharides stimulated the extracellular signal-regulated protein kinase (ERK) / cyclic AMP-responsive element-binding protein (CREB) signaling pathway, amplifying the expression of brain-derived neurotrophic factor (BDNF) and contributing to neuronal survival and regeneration (43).

Emerging evidence has suggested that metabolic dysregulations aggravate the occurrence and development of AD, around 80% of AD patients exhibit insulin resistance, which some scholars refer to as type III diabetes (54). In light of insulin-related signaling’s importance in energy homeostasis, neuronal survival, and memory processes, and the fact that insulin resistance is associated with memory impairment and other AD symptoms, substances that modulate insulin signaling should be considered as potential AD treatments (55). Notably, administering okra polysaccharides (56), yeast β-glucan (57), and *Astragalus membranaceus* polysaccharides (42) had shown effectiveness in alleviating insulin resistance and reducing cognitive impairment in AD model mice. Additionally, disturbances in amino acid metabolism can cause neurotoxicity, affecting neurotransmitter function, cognition, and emotional regulation, worsening neurodegeneration (58). Yet, treatment with *Cistanche deserticola* polysaccharides restored valine, L-methionine, uric acid and proline levels in serum, alleviated an amino acid imbalance, and enhanced cognitive function in D-galactose-induced AD mice (37). Furthermore, cholinergic metabolism, crucial for regulating neurotransmission, memory, and muscle movement, might also contribute to AD-related cognitive decline, possibly linked to abnormal cholinergic neuron count and function (59). Encouragingly, Angelica sinensis polysaccharides exhibited promise in reducing acetylcholinesterase (AChE) levels, elevating acetylcholine (ACh) and choline acetyltransferase (ChAT) levels, and improving memory impairment in AD rats through the BDNF/CREB pathway (43). Moreover, the hypothalamic–pituitary–adrenal (HPA) axis, also a key stress response component, significantly influences AD progression (60). These findings underscore the crucial roles of glucose and amino acid metabolism, cholinergic regulation, and HPA axis function in AD pathogenesis while highlighting the therapeutic potential of dietary polysaccharides in addressing AD-associated pathological processes.

Additionally, studies have found that the gut microbiota can influence the occurrence of AD through various pathways mediated by the gut-brain axis (61). Microbiota dysbiosis enhances immuno-senescence, oxidative stress, cytokine secretion, and neuroinflammation, which are involved in the early disease stages of AD (62). Studies have shown that *Anoectochilus roxburghii (Wall.) Lindl.* polysaccharides (63), *Cistanche deserticola* polysaccharides (37), inulin (64), fructan (65), and yeast β-glucan (57) could mitigate cognitive deficits and mental disorders by enriching beneficial bacteria, decreasing pathogenic bacteria, restoring the intestinal epithelial barrier, and augmenting SCFAs.

These findings collectively highlight those dietary polysaccharides are expected to intervene in the development and progression of AD by alleviating oxidative stress, neuroinflammation, metabolic dysregulation, and gut microbiota disorders (Table 2), presenting a promising direction for future therapeutic strategies.

**Table 2:** Recent studies on neuroprotective effects of dietary polysaccharides on AD model.

Type of dietary polysaccharides	Models/methods	Protective effects	Authors, references
Chondroitin sulfate E from squid cartilage	*C. elegans* strains / 0.25, 0.5, 1 mg/mL added in the Nematode growth media culture plates	Chondroitin sulfate E reduced oxidative stress and suppressed Aβ deposition, alleviated Aβ-induced worm paralysis and chemotaxis dysfunction in transgenic *C. elegans*.	Wang et al. (47)
*Anoectochilus roxburghii (Wall.) Lindl.* polysaccharides	6-week-old male C57BL/6 J mice / 1 g/kg and 3 g/kg orally administered for 14 weeks	*Anoectochilus roxburghii (Wall.) Lindl.* polysaccharides ameliorated memory and cognitive impairment in obese mice by improving neuroinflammation. *Anoectochilus roxburghii (Wall.) Lindl.* polysaccharides treatment also restored the intestinal epithelial barrier by upregulating intestinal tight junction proteins.	Fu et al. (63)
Fructan	1,837 elderly people (≥65 years) / no additional additions	Higher fructan intake is associated with reduced risk of clinical AD among older adults.	Nishikawa et al. (65)
Low molecular weight chondroitin sulfate from shark cartilage	Male 5XFAD mice / 50, 150, 450 mg/kg orally administered for 3 months	Low molecular weight chondroitin sulfate administration ameliorated APP metabolism, neuroinflammation, ROS production and tau protein abnormality in the brains of 5XFAD mice, displaying the potential to improve the pathological changes of AD mouse brain.	Zhao et al. (48)
*Cistanche deserticola* polysaccharides	8-week-old male Kunming mice / 25, 50, 100 mg/kg orally administered for 2 months	*Cistanche deserticola* polysaccharides treatment improved cognitive function, restored gut microbial homeostasis, thereby reducing oxidative stress and peripheral in D-galactose-treated mice	Gao et al. (37)
Inulin	3-month-old E3FAD and E4FAD mice / 8% inulin in the diet for 16 weeks	Early inulin interventions improved brain and systemic metabolism via enhancing the gut-brain axis, in both E3FAD and E4FAD mice.	Yanckello et al. (64)
*Poria cocos* polysaccharides	Male Wister rats /100, 200 and 300 mg/kg intragastric administration for 30 days	*Poria cocos* polysaccharides prevented cognitive decline, reduced neuronal apoptosis in hippocampus, alleviated oxidative stress, inflammation and inhibited the MAPK/NF-κB pathway in rats AD model.	Zhou et al. (38)
Non-saponin fraction with rich polysaccharides from Korean red ginseng	5.5-month-old male 5 × FAD male mice / 150 mg/kg intragastric administration for 8 weeks	Polysaccharides from Korean red ginseng treatment ameliorated Aβ-induced cognitive impairment in 5 × FAD mice, alleviated Aβ deposition, neuroinflammation, neurodegeneration, mitochondrial dysfunction, and impairment of adult hippocampal neurogenesis both *in vivo* and *in vitro*.	Shin et al. (39)
Okra polysaccharides	12-week-old male Kunming mice / 300 mg/kg and 600 mg/kg oral administrated for 8 weeks	Okra polysaccharides treatment reversed the metabolic disorder induced by high-fat diet and cognitive function injury in AD model mice.	Yan et al. (56)
Yeast β-glucan	8-week-old male C57BL/6 J mice / 100 mg/kg oral administrated for 4 weeks	Yeast β-glucan ameliorated cognition deficits and pathological changes through gut-brain axis and alleviating brain inflammation in AD-like mice.	Xu et al. (57)
Polysaccharides of *Taxus chinensis* var. *mairei* Cheng et L.K.Fu	8-week-old male C57BL/6 mice / 0.4 g/kg intragastric administration for 14 days	Polysaccharides of *Taxus chinensis var. mairei* Cheng et L.K.Fu administration restored the impaired learning and cognitive function in mice AD model, inhibited Aβ deposition, apoptosis and oxidative stress in BV2 cells induced by D-gal.	Zhang et al. (40)
astragalus polysaccharidea	7-month-old male APP/PS1 mice / 200 mg/kg oral administrated for 2 months	Astragalus polysaccharides treatment increased the expression of Nrf2 in the nucleus, restored the expression levels of Keap1, SOD, glutathione peroxidase (GSH-Px) and MDA, improved the cognitive ability, reduced apoptosis and the accumulation of Aβ in APP/PS1 mice.	Qin et al. (41)
Polysaccharides from *Lycium barbarum*	7-month-old male APP/PS1 mice / 50 mg/kg oral administrated for 3 months	*Lycium barbarum* polysaccharides treatment enhanced neurogenesis and restored synaptic dysfunction in hippocampus CA3-CA1 area, reduced Aβ level and improve the cognitive functions in APP/PS1 mice.	Zhou et al. (51)
*Alpinia oxyphylla* crude polysaccharides	6-week-old male Kungming mice / 500 mg/kg oral administrated for 2 weeks	*Alpinia oxyphylla* crude polysaccharides treatment improved learning and memory ability in AD mice, restored normal levels of NO, IL-1β, PGE-2, and TNF-α in the serum of AD mice.	Shi et al. (49)
*Codonopsis pilosula* polysaccharides	5.5-month-old male APP/PS1 mice / 100 mg/kg and 300 mg/kg oral administrated for 1 months	*Codonopsis pilosula* polysaccharides ameliorated cognitive defects in APP/PS1 mice, and inhibited BACE1 activity in cultured cells.	Wan et al. (53)
*Angelica sinensis* polysaccharides	male SD rats / 50 mg/kg oral administrated for 4 weeks	*Angelica sinensis* polysaccharides treatment ameliorated memory impairment, regulated the balance of neurotransmitters, free radical metabolism, and inflammation, activated the BDNF/TrkB/CREB pathway in Aβ_25–35_-induced AD rats.	Du et al. (43)
*Schisandra* polysaccharides	6-week-old male SD rats / 38.15 mg/kg oral administrated for 56 days	*Schisandra* polysaccharides significantly improved the memory acquisition ability and reversed the memory consolidation disorder of the AD rats inhibiting Aβ formation, tau protein phosphorylation and antioxidative damage.	Liu et al. (44)
Polysaccharides of *Schisandra Chinensis* Fructus	Male KM mice / 260 mg/kg oral administrated for 28 days	Polysaccharides of *Schisandra Chinensis* Fructus improved the cognition and histopathological changes, reduced the deposition of Aβ, downregulated the expression of pro-inflammatory cytokines and activated the NF-κB/MAPK pathway in AD mice.	Xu et al. (50)
*Inonotus obliquus* polysaccharides	8-month-old male APP mice / 25 and 50 mg/kg oral administrated for 8 weeks	*Inonotus obliquus* polysaccharides improved the pathological behaviors related to memory and cognition, reduced the deposition of β-amyloid peptides and neuronal fiber tangles in the brain, and modulated the levels of anti- and pro-oxidative stress enzymes, enhanced the expression levels of Nrf2 and its downstream proteins, including Heme Oxygenase-1 (HO-1) and SOD-1, in the brains of APP/PS1 mice.	Han et al. (45)
*Flammulina velutipes* polysaccharides	male Wistar AD rats / 200 or 400 mg/kg oral administrated for 30 days	*Flammulina velutipes* polysaccharides and ginsenosides treatment elevated cognitive ability, lowered the Bax/Bcl-2 ratio, processed the anti-oxidant and anti-apoptosis effects in AD rats.	Zhang et al. (46)
*Astragalus membranaceus* polysaccharides	APP/PS1 mice/ 500 mg/kg oral administrated for 7 weeks	*Astragalus membranaceus* polysaccharides administration reduced metabolic stress-induced increase of body weight, insulin and leptin level, insulin resistance, and hepatic triglyceride, ameliorated metabolic stress-exacerbated oral glucose intolerance, diminished metabolic stress-elicited astrogliosis and microglia activation in the vicinity of plaques in brain.	Huang et al. (42)
*Ganoderma lucidum* polysaccharides	6-month-old APP/PS1 mice / 30 mg/kg oral administrated for 14 days	*Ganoderma lucidum* polysaccharides promoted neural progenitor cell proliferation to enhance neurogenesis and alleviated cognitive deficits in transgenic AD mice, promoted self-renewal of neural progenitor cell, enhanced the activation of FGFR1 and its downstream ERK and AKT cascades *in vitro*.	Huang et al. (52)

## 5 Dietary polysaccharides and the interventions for depression and anxiety disorder

As a common psychiatric disorder, depression is characterized by feelings of sadness, guilt, and lack of interest in and self-worth; tiredness; poor concentration; poor appetite, and disturbed sleep (66). In 2017, the WHO reported that approximately 4.4% of the global population suffers from depression, and depression has become the leading contributor to suicide attempts (66). Anxiety disorder, another most common psychiatric disorder, is characterized by symptoms such as nervousness, restlessness, and vegetative dysfunction without a specific trigger (67). Up to 33.7% of the population suffers from anxiety disorders during their lifetime, and anxiety disorders are prone to combine with other mental disorders (67). Although depression and anxiety are distinct emotional states, they often co-occur and are highly comorbidity (68). Data suggests that approximately 85% of patients with depression also experience significant anxiety, while 90% of individuals with anxiety disorders also have symptoms of depression. Recent evidence suggests genetic and neurobiological similarities between depressive and anxiety disorders (69).

Due to its comparatively high oxygen utilization and lipid-rich constitution, the brain is considered particularly vulnerable to oxidative damage (70). Together with the pathological changes associated with many psychiatric syndromes, this intrinsic oxidative vulnerability suggests that oxidative damage could be a plausible pathogenic candidate for anxiety and depression (70). Oxidative stress-induced neuroinflammation not only affects individual neurons but also reaches synaptic connections, known as synapses (71). Abnormal structure and function of synapses, affect the communication between neurons and the balance of neurotransmitters, which contribute to the development and progression of depressive and anxious symptoms (72). However, dietary polysaccharides have demonstrated beneficial effects in improving depression and anxiety, with their antioxidative and anti-inflammatory properties playing significant roles. Administration of acidic polysaccharides from poria (73), inulin (74, 75), *Polygonatum sibiricum* F. Delaroche polysaccharides (76), *Ganoderma lucidum* polysaccharides (77), polysaccharide from okra (*Abelmoschus esculentus* (L) Moench) (78), ameliorated anxiety disorders and depressive behaviors, regulated the levels of multiple factors related to oxidative stress, reduced proinflammatory cytokine levels. At the same time, all the dietary polysaccharides mentioned above provided protective effects on neurons, such as reducing synaptic damage, enhancing synaptic activity, and regulating the expression of synapse-related proteins and genes. In addition, *Lycium Barbarum* polysaccharides (79) also alleviated the depression-like and social anxiety-like behavior by enhancing synaptic plasticity and maintaining the normal function of synapses.

The HPA axis is a crucial component of the neuroendocrine system. It becomes intensified in response to external stimuli, leading to the secretion of corticosterone by the adrenal glands, which helps the body adapt to the new environment (80). Hyperfunction of the HPA axis is considered an important factor in the development of depression and anxiety (81), although the regulation of other peptides or hormones within the HPA axis may differ between these two disorders (82). In addition, the intimate connection between the HPA axis and neurotransmitters also regulates the mood, cognition, and behavior in depression and anxiety patients (83, 84). Studies have shown that *Polygonatum sibiricum* F. Delaroche polysaccharides (76), inhibited the hyperfunctioning of the HPA axis. Moreover, partially hydrolyzed guar gum (85), acidic polysaccharides from poria (73), *Polygonatum sibiricum* F. Delaroche polysaccharides (76), total polysaccharides of *Lilium lancifolium* Thunberg (86), and polysaccharide from *Ginkgo biloba* leaves (87), regulated the neurotransmitter levels in multiple brain regions in rodent models of depression and anxiety.

There is increasing evidence that gut microbiota is associated with anxiety and depression. Although diversity findings were inconsistent, specific bacterial taxa were implicated according to clinical research findings: higher abundance of proinflammatory species (e.g., *Enterobacteriaceae* and *Desulfovibrio*), and lower SCFAs producing-bacteria (e.g., *Faecalibacterium*) in patients with anxiety/depressive disorders (88). An analysis of the composition of gut microbiota suggested that polysaccharides from okra (*Abelmoschus esculentus* (L) Moench) decreased the relative proportions of *Bacteroidetes* and *Actinobacteria*, while increasing *Firmicutes* at the phylum level in chronic unpredictable mild stress (CUMS)-induced depression mice. Simultaneously, the generation of SCFAs were also found to contribute positively to the antidepressant-like effect (78). Administration of partially hydrolyzed guar gum (85), inulin (74, 75), polydextrose (89), polysaccharide from *Ginkgo biloba* leaves (87), 3’Sialyllactose and 6’Sialyllactose (90) also had certain regulatory effects on the gut microbiota in rodent models of depression and anxiety.

The above studies demonstrate that polysaccharide compounds can improve and alleviate depression and anxiety disorders (Table 3). Nevertheless, the mechanisms by which dietary polysaccharides regulate the expression of synapse-associated proteins, reduce synaptic damage, and regulate the gut microbiota to prevent depression and anxiety need further research. In addition, the efficacy of dietary polysaccharides brought into the daily diet requires to be evaluated in further clinical trials.

**Table 3:** Recent studies on neuroprotective effects of dietary polysaccharides on depression and anxiety disorder models.

Type of dietary polysaccharides	Models/methods	Protective effects	Authors, references
Partially hydrolyzed guar gum	5-week-old male C57BL/6 mice / 600 mg/kg oral administration for 28 days	Partially hydrolyzed guar gum administration inhibited the loss of body weight, prevented CUMS-induced depressive-like behavior, improved the species richness and diversity of gut microbiota in depression model mice.	Chen et al. (85)
Acidic polysaccharides from poria	Male SD rats / 100, 300, and 500 mg/kg oral administration	Treatment of acidic polysaccharides from poria improved the depression-like behavior, increased the number of neurons and the levels of neurotransmitters in the hippocampus, regulated NLRP3 inflammasome signaling pathway in depression model rats.	Chen et al. (73)
Inulin	6-week-old male C57BL/6 J mice / 2 g/kg oral administration for 6 weeks	Iulin administration ameliorated anxiety disorders and depressive behaviors, reduced neuroinflammation and neuronal damage, improved intestinal integrity and permeability, modulated the gut microbiota in schizophrenia model mice.	Guo et al. (75)
*Polygonatum sibiricum* F. Delaroche polysaccharides	3-month-old male C57BL/6 mice / 100, 200, and 400 mg/kg intragastric administration for 10 days	*Polygonatum sibiricum* F. Delaroche polysaccharides prevented depression-like behaviors, increased the serotonin level and ameliorated hippocampal synaptic and cellular injury, reduced the inflammatory response, and eliminated ROS and HPA axis hyperfunction in lipopolysaccharide (LPS)-treated and CUMS mice models.	Shen et al. (76)
*Ganoderma lucidum* polysaccharides	7-8-week-old male C57BL/6 mice /1 mg/kg, 5 mg/kg, and 12.5 mg/kg intraperitoneal administration for 5 days	*Ganoderma lucidum* polysaccharides treatment enhanced anti-inflammatory neuroimmune status and synaptic plasticity, led to antidepressant effects on chronic social defeat stress mice via modulation of Dectin-1.	Li et al. (77)
Polysaccharide from okra (*Abelmoschus esculentus* (L) Moench)	Male C57BL/6 mice / 400 mg/kg oral administration for 2 weeks	Polysaccharide from okra treatment alleviated depressive and anxiety behavior in CUMS mice model, reduced the rising proinflammatory cytokines in the colon, serum, and hippocampus, regulated the gut microbiota profiles and composition, reversed Toll-like receptor 4 (TLR4)/ nuclear factor-kappa B (NF-κB) and mitogen-activated protein kinases (MAPKs) signaling in hippocampus.	Yan et al. (78)
Polydextrose	Healthy female / 12.5 g for 4 weeks	Polydextrose supplementation improved cognitive flexibility, increased the abundance of *Ruminiclostridium*, although there was no change in microbial diversity, attenuated the expression of adhesion receptor CD62L receptor, a marker of acute stress responsiveness.	Berding et al. (89)
Total polysaccharides of *Lilium lancifolium* Thunberg	8-week-old female C57BL/6 N mice / 50, 100, and 200 mg/kg intragastric administration for 36 days	Total polysaccharides of lily bulb showed positive effects in reducing ovariectomized-induced anxiety, depression, and cognitive impairment, triggered the specific Ras/Akt/ERK/CREB signaling pathway, and modulated multiple proteins associated with mitochondrial oxidative stress. The potential mechanism was more closely associated with the predominant activation of estrogen receptors and regulation of brain regional neurotransmitters and neurotrophins with minor effects on the uterus.	Zhou et al. (86)
Polysaccharide from *Ginkgo biloba* leaves	3-4-week-old male BALB/c mice/ 300 mg/kg intragastric administration for 30 days	Polysaccharide from *Ginkgo biloba* leaves administration reduced the stress-induced depression, elevated serotonin and dopamine levels in multiple brain regions including the hippocampus, cerebral cortex and olfactory bulb, reversed gut dysbiosis and increased the richness of *Lactobacillus* species.	Chen et al. (87)
*Lycium barbarum* polysaccharides	7-8-week-old male SD rats / 1 mg/kg intragastric administration for 14 days	*Lycium barbarum* polysaccharides alleviated the depression-like and social anxiety-like behaviors in rats and restored the hippocampal neurogenesis after the dextromethorphan treatment.	Po et al. (79)
Inulin from yacon	Male Kuming mice and SD rats / 25, 50, or 100 mg/kg intragastric administration for 5 days	Inulin extracted from yacon treatment reduced the immobility time in the mouse tail suspension test and the forced swimming test, reversed the escape deficits in learned helplessness rats.	An et al. (74)
3’Sialyllactose and 6’Sialyllactose	6–8-week-old male C57/BL6 mice / 5% of the diet by oral administration for 2 weeks	3′Sialyllactose or 6′Sialyllactose helped maintain normal behavior on tests of anxiety-like behavior and normal numbers of doublecortin^+^ immature neurons.	Tarr et al. (90)

## 6 Dietary polysaccharides and the interventions for autism spectrum disorder

Autism spectrum disorder (ASD) is a neurodevelopmental disorder believed to be caused by early brain changes and neuronal reorganization. Individuals with ASD exhibit a wide range of symptoms, but they share a common set of disorders, including impairments in social communication and repetitive sensory-motor behaviors (91). The average prevalence of ASD in Asia, Europe, and North America is estimated to be approximately 1%, males are more affected by autism than females, and comorbidity is common (more than 70% of people with autism have concurrent conditions) (92).

To date, the exact underlying causes of ASD are not yet fully understood. Clinical studies have shown that gastrointestinal symptoms such as constipation, diarrhea and gut microbiota imbalance are common in ASD patients (93). Unfortunately, there are no effective treatments for the core symptoms of ASD (94). Statistics show that approximately 50–70% of ASD patients resort to bio-related therapies such as antibiotics, antifungal and antiviral drugs, gastrointestinal medications, nutritional supplement therapy, and restrictive or special diets. However, most of these interventions lack comprehensive safety and efficacy assessments (94). With the deepening research on the relationship between human microbiome and ASD, scientists have gradually realized the importance of gut microbiota in affecting neurodevelopment and brain function. Partially hydrolyzed guar gum, a form of prebiotic dietary water-soluble fiber, has been shown to modulate the gut microbiota and stimulate the production of SCFAs in healthy adults. Moreover, the supplementation with partially hydrolyzed guar gum resulted in a trend toward decreased serum level of Interleukin-6 (IL-6) and TNF-α, which help improve behavioral irritability and constipation of children with ASD (95). Hence, polysaccharide intervention seems to help in the effective treatment of ASD. However, the mechanism of dietary polysaccharides to relieve constipation and gut microbiota dysbiosis caused by ASD also needs further study. Moreover, there are fewer dietary polysaccharides that can interfere with ASD, and it is the direction of our further research to find more dietary polysaccharides to treat children with ASD.

## 7 Dietary polysaccharides and the interventions for epilepsy

Epilepsy is a chronic non-communicable neurological dysfunctions disorder. It is primarily characterized by seizures, repetition, stereotypy, and transience, which belong to an involuntary movement involving a portion or the whole body, sometimes combined with the loss of consciousness and control of bowel or bladder function (96). Epilepsy can affect individuals of all ages, genders, races, income groups, and geographical regions (97). According to the WHO, approximately 5 million people worldwide are diagnosed with epilepsy each year. As of 2019, China alone had over 9 million people living with epilepsy, and the number of new cases continues to rise (98).

Seizures in epilepsy can be triggered by various factors that disrupt normal neuron activity, including illnesses, brain damage, abnormal brain development, imbalances in neurotransmitters, changes in ion channels, or a combination of these and other factors (99). Mutations in genes related to ion channels have been strongly linked to seizures, especially calcium ion channels (100). The effects of Ca^2+^ are typically mediated through its interaction with calmodulin (CaM) (101), epileptogenic factors can downregulate CaM, leading to increased neuronal activity and the development of epilepsy. CaM may also indirectly contribute to the pathological process of epilepsy by modulating calcium/CaM-dependent protein kinases (CaMK) (102). Studies have shown that *Ganoderma lucidum* polysaccharides inhibited the Ca^2+^ accumulation in neurons and subsequent stimulation of CaMK II α expression, which indicates a beneficial role in the prevention or treatment of epilepsy (103).

Mitochondrial dysfunction and oxidative stress have also been considered potential causes of epileptic seizures. Seizures can trigger neuroinflammatory responses that further directly impact the electrical activity of neurons and glial cells, and exacerbate CNS damage, forming the pathological basis for refractory epilepsy (104). Dietary polysaccharides have shown potential in reducing inflammatory responses, inhibiting the expression of neurotransmission-related genes, and improving hippocampal tissue damage in epilepsy models. Due to the antioxidant and anti-inflammatory properties of chondroitin sulfate, a significant reduction in seizure mice induced by pentylenetetrazole and pilocarpine was observed (105). Fructus corni polysaccharide treatment (106) decreased levels of ROS and malondialdehyde (MDA), increased superoxide dismutase (SOD) activity, and inhibited expressions of phosphorylated-Jun N-terminal Kinase (p-JNK), cytochrome C, and caspase-3 in an epileptic rat model. Angelica polysaccharide promoted cell proliferation, inhibited apoptosis, and suppressed IL-1β, TNF-α, and IL-6 production in epilepsy cell models (107). Thus, dietary polysaccharides intervention to improve epilepsy focuses on inflammatory response, oxidative stress, and ion channels and signaling pathways regulated by related genes (Table 4). In the future, combined with microbial studies, it is possible to explore whether dietary polysaccharides show their functional activities by regulating gut microbiota and intestinal microecology.

**Table 4:** Recent studies on neuroprotective effects of dietary polysaccharides on epilepsy model.

Type of dietary polysaccharides	Models/methods	Protective effects	Authors, references
Chondroitin sulfate	Male Swiss albino mice / 100, 200 mg/kg intragastric administration for 15 days	Administration of chondroitin sulfate concluded a significant and dose-dependent attenuation of pentylenetetrazole- and pilocarpine-induced seizures in mice. Additionally, chondroitin sulfate suppressed levels of caspase-3, showed its antioxidant and anti-inflammatory properties, indicating a neuroprotective treatment strategy in epilepsy.	Singh et al. (105)
Angelica polysaccharide	Mouse hippocampal neuronal HT22 cells / 0 to 100 μg/mL add io the culture medium	Angelica polysaccharide mitigated LPS-evoked inflammatory injury through repression of NF-κB and JAK2/STAT3 pathways by regulating miR-10a in HT22 cells. The discoveries offered a novel strategy for the clinical remedy of epilepsy.	Zhou et al. (107)
Fructus corni polysaccharide	6-8-week-old male SD rats / 100, 200, 300 mg/kg intragastric administration for 24 days	Fructus corni polysaccharide decreased the alteration in ΔΨm, cytochrome C leakage, and the activation of cleaved caspase-3 by reducing the activation of hippocampus ROS and the MAPK cascade pathway following epilepsy, thereby alleviating the apoptosis of neurons and having a neuroprotective effect on epilepsy.	Sun et al. (106)
*Ganoderma lucidum* polysaccharides	Primary hippocampal neurons from newborn Wistar rats / 0.375 mg/mL add in the culture medium	*Ganoderma lucidum* polysaccharides inhibited the Ca^2+^ accumulation in neurons and subsequent stimulation of CaMK II α expression, which indicates a beneficial role in the prevention or treatment of epilepsy.	Wang et al. (103)

## 8 Dietary polysaccharides and the interventions for stroke

Stroke, an acute cerebrovascular disease, is another neurological disorder. About 80–85% of strokes are ischemic (the blockage of blood vessels preventing blood from flowing to the brain) (108), while about 15–20% are hemorrhagic (the sudden rupture of blood vessels in the brain) (109). The clinical manifestations of stroke include sudden weakness on one side of the body, fainting, unconsciousness, confusion, difficulty speaking or understanding, vision problems, and loss of balance or coordination (110). Over the past 20 years, the global burden of stroke has increased significantly, with an increase of 70.0% in incident strokes, 43.0% in deaths caused by stroke, 102.0% in prevalent strokes, and 143.0% in disability-adjusted life years (111). The prevalence of stroke in northern China in 2022 has nearly doubled compared to 2010 (112).

Stroke is a significant contributor to premature mortality, yet there is no effective treatment for stroke to improve blood circulation in the affected brain area and restore neurological function (113). Clinically, stroke symptoms are alleviated with medications targeting neuroprotection and cerebrovascular circulation enhancement. However, prolonged use of these drugs may adversely affect liver and kidney function, further impairing overall organ function (110). Consequently, dietary polysaccharides and their derivatives have been studied for their potential to alleviate the effects of stroke. *Lycium barbarum* polysaccharides exerted a neuroprotective effect against ischemic injury through dual actions of activating the N-methyl-D-aspartic acid receptor subunit 2A (NR2A) signaling pathway and inhibiting the N-methyl-D-aspartic acid receptor subunit 2B (NR2B) signaling pathway. This effect reduced the death of CA1 neurons after transient global cerebral ischemia and improved memory impairment in ischemic rats (114). Additionally, *Momordica charantia* polysaccharides provided neuroprotection against cerebral ischemia/reperfusion injury by inhibiting lipid peroxidation and preserving antioxidant enzyme activity (115). Furthermore, the neuroprotective effect of *Ginkgo biloba* polysaccharide is achieved by suppressing oxidative stress and reducing the concentration of inflammatory factors. This action decreased the cerebral infarction area in rats and ameliorates neurofunctional deficits (116). Hence, dietary polysaccharides play a beneficial role in ischemic stroke mainly by alleviating oxidative stress, reducing inflammatory factors, and promoting neuronal cell repair. These effects of dietary polysaccharides also suggest that the gut microbiota might play a crucial role in stroke, however, the exact impact of gut microbiota on stroke remains to be further studied. Table 5 provides examples of studies that have explored the use of dietary polysaccharides in improving cerebral stroke.

**Table 5:** Recent studies on neuroprotective effects of dietary polysaccharides on stroke model.

Type of dietary polysaccharides	Models/methods	Protective effects	Authors, references
*Lycium barbarum* Polysaccharides	Adult male Wister rats / 20 mg/kg intragastric administration for 1–2 weeks	*Lycium barbarum* polysaccharides reduced CA1 neurons from death after transient global ischemia and ameliorated memory deficit in ischemic rats, activated the NR2A-mediated survival pathway and inhibited the NR2B-mediated apoptotic pathway in primary cultured cortical neurons, which suggests that *Lycium barbarum* Polysaccharides may be a superior therapeutic candidate for the treatment of ischemic stroke.	Shi et al. (114)
*Momordica charantia* polysaccharides	Adult male SD rats / 50, 100, 200 mg/kg intraperitoneally at 30 min prior or after cerebral ischemia	*Momordica charantia* polysaccharides dose-dependently attenuated apoptotic cell death in neural cells under oxygen glucose deprivation condition *in vitro* and reduced infarction volume in ischemic brains *in vivo*; inhibited lipid peroxidation, and inhibited the JNK3 signaling cascades during cerebral ischemia/reperfusion injury.	Gong et al. (115)
*Ginkgo biloba* polysaccharide	Male SD rats / 100, 200, 400 g/kg oral administration for 7 days	Treatment of *Ginkgo biloba* polysaccharide before focal ischemia/reperfusion injury decreased cerebral infarct size and improved neurological deficits in rats, and the neuroprotective effects were mediated by suppression of NO production, decreased concentrations of TNF-α and IL-1β, increased concentration of IL-10, and inhibition of oxidative stress as evidenced by increased SOD activity and decreased MDA level.	Yang et al. (116)

## 9 Remarks

**Figure 1:**
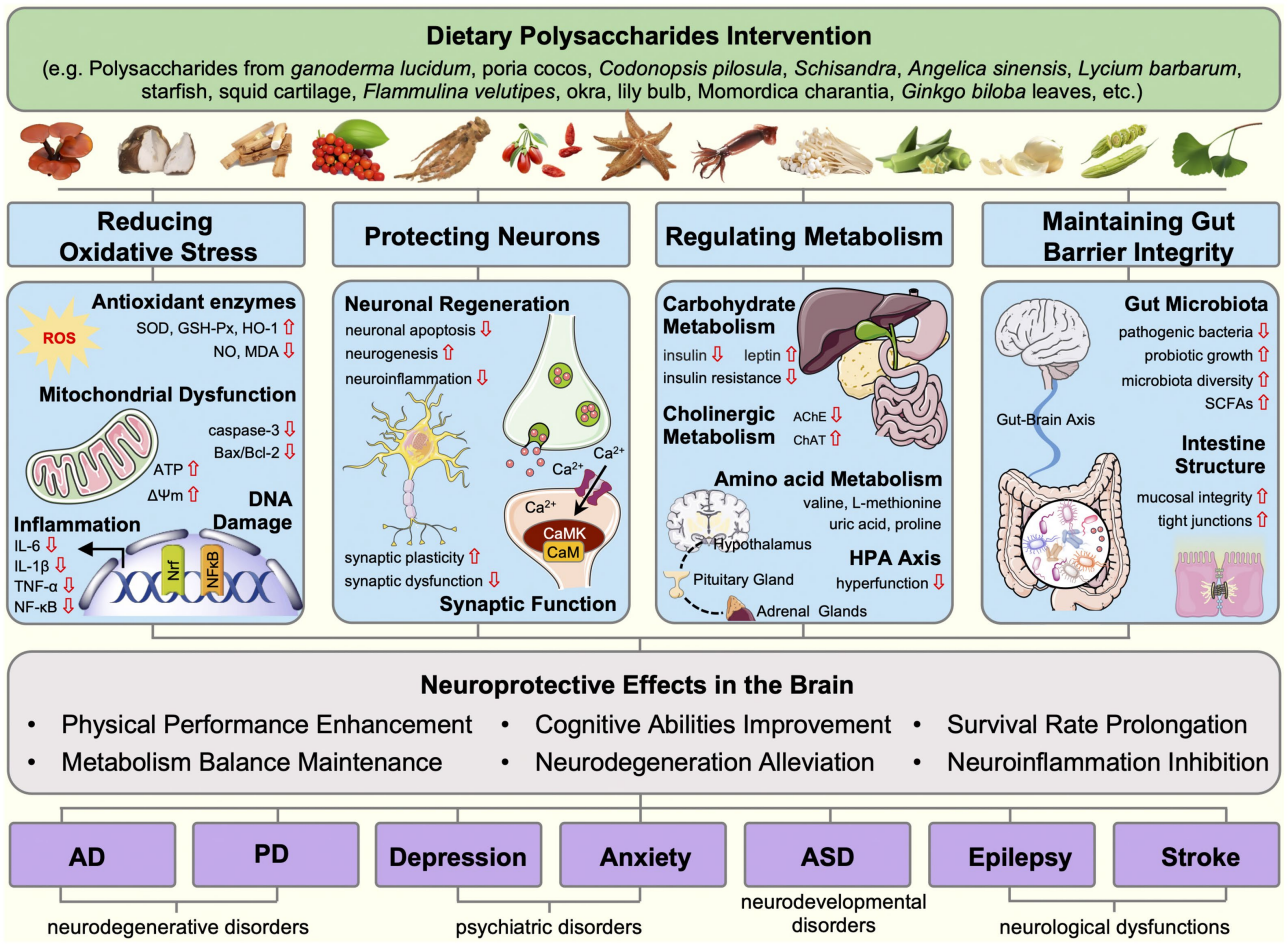
Mechanisms linking dietary polysaccharides and CNS diseases, Interventions by dietary polysaccharides in neurodegenerative diseases (Alzheimer’s disease, and Parkinson’s disease), mental disorders (depression and anxiety), neurodevelopmental disorders (autism spectrum disorders), and neurological dysfunctions (epilepsy and stroke) can be categorized into four mechanistic foundations, including the oxidative stress reduction, neuronal protection, metabolism regulation, and gut barrier integrity maintenance. Dietary polysaccharides reduce oxidative stress by enhancing antioxidant enzyme activity, restoring mitochondrial function, and reducing the production of inflammatory factors. Dietary polysaccharides restore the normal function of neurons by increasing neurogenesis, alleviating neuroinflammation, regulating ion channels, and enhancing synaptic plasticity. Dietary polysaccharides maintain metabolic balance and promote brain health by modulating carbohydrate metabolism, cholinergic metabolism, amino acid metabolism, HPA axis, and the neurotransmitter system. Furthermore, dietary polysaccharides exert neuroprotective effects by regulating gut microbiota and their metabolite composition, preserving gut mucosal barrier integrity, and influencing gut-brain communication.

In conclusion, the review sheds light on the role of dietary polysaccharides in neurodegenerative disorders, psychiatric disorders, neurodevelopmental disorders, and neurological dysfunctions. The potential mechanisms of dietary polysaccharides involved ameliorating oxidative stress, neuronal injury, metabolic abnormalities, and gut microbiota disorder (Figure 1). Multifaceted effects of dietary polysaccharides on these diseases are noteworthy, with a common thread being their antioxidant activity and gut microbiota regulation.

In models of neurodegenerative diseases, such as PD or AD, dietary polysaccharides have been shown to provide neuroprotective effects by decreasing oxidative stress and modulating the gut microbiota. Dietary polysaccharides are found to reduce oxidative stress and inflammation, regulate the HPA axis and neurotransmitter system, and maintain gut microbiota balance in psychiatric disorders, such as anxiety and depression. By regulating the gut microbiota, dietary polysaccharides may also influence neurodevelopmental disorders (like ASD). In addition, dietary polysaccharides exert antiepileptic effects by controlling ion channels, reducing oxidative stress and neuroinflammation, and restoring mitochondrial function. The neuroprotective properties of dietary polysaccharides in the context of stroke further highlight their role in protecting neurons by inhibiting oxidative stress and anti-inflammatory mechanisms. Together, these comprehensive findings emphasize the critical role of oxidative stress as a key factor in the development of CNS diseases. Dietary polysaccharides are potential therapeutic agents for alleviating these diseases due to their outstanding antioxidant and anti-inflammatory properties. Notably, dietary polysaccharides can also exert beneficial effects by regulating the composition of gut microbiota and its metabolites, protecting the integrity of the intestinal mucosal barrier, and affecting gut-brain communication.

However, it is imperative to acknowledge that despite the range of diseases examined in this study, the full spectrum of CNS disorders susceptible to polysaccharide regulation remains incompletely explored, underscoring the need for further research initiatives. Additionally, this paper falls short of clarifying which specific components and structures in dietary polysaccharides work and how the metabolic fragments of dietary polysaccharides influence the organism. Moreover, it is crucial to recognize that a substantial portion of the studies reviewed are confined to *in vitro* or animal experimentation. Despite the intake of dietary polysaccharides from natural sources in the normal diet is generally recognized as safe (GRAS), a series of clinical trials is still imperative to determine the lowest effective dosage in human and to minimize the side effects. It is possible to convert the dosage between animals and human by body surface area or body weight, considering the appropriate dosage and safety of dietary polysaccharides in human beings (117). Thereby, advanced preclinical and clinical investigations are needed to substantiate the translational potential of dietary polysaccharides as intervention candidates for CNS disorders. Consequently, a more comprehensive understanding of dietary polysaccharides’ true potential in promoting CNS health hinges on a broader repertoire of research endeavors, including more extensive preclinical evaluations and compelling clinical trials. These collective endeavors are poised to shed light on the genuine capabilities of dietary polysaccharides in CNS health and provide a robust platform for the development of novel therapeutic strategies.

## Author contributions

RG: Conceptualization, Writing – original draft. JP: Conceptualization, Writing – original draft. JZ: Conceptualization, Writing – original draft. XX: Writing – review & editing. JL: Writing – review & editing. JML: Writing – review & editing. WW: Writing – original draft. SZ: Writing – original draft. YZ: Writing – original draft. ZZ: Writing – review & editing. HC: Writing – review & editing. TY: Conceptualization, Writing – review & editing. SW: Writing – review & editing. ZL: Conceptualization, Writing – review & editing.

## Glossary

Ach: acetylcholine
AChE: acetylcholinesterase
AD: Alzheimer’s disease
ASD: autism spectrum disorder
Aβ: β-like amyloid
BDNF: brain-derived neurotrophic factor
CaM: calmodulin
CaMK: CaM-dependent protein kinases
ChAT: choline acetyltransferase
CNS: central nervous system
CREB: cyclic AMP-responsive element-binding protein
CUMS: chronic unpredictable mild stress
ERK: extracellular signal-regulated protein kinase
GSH-Px: glutathione peroxidase
HPA: hypothalamic–pituitary–adrenal
IL-1β: Interleukin-1 beta
LPS: lipopolysaccharide
MDA: malondialdehyde
NFTs: neuronal fibrillary tangles
NR2A: N-methyl-D-aspartic acid receptor subunit 2A
NR2B: N-methyl-D-aspartic acid receptor subunit 2B
Nrf2: nuclear transcription factor 2
PD: Parkinson’s disease
PGC-1α: peroxisome proliferator-activated receptor gamma coactivator 1-alpha
p-JNK: phosphorylated-Jun N-terminal Kinase
ROS: reactive oxygen species
SCFAs: short-chain fatty acids
SOD: superoxide dismutase
TNF-α: Tumor Necrosis Factor-alpha
WHO: World Health Organization
ΔΨm: mitochondrial membrane potential
